# The *Arabidopsis* Cytosolic Ribosomal Proteome: From form to Function

**DOI:** 10.3389/fpls.2013.00032

**Published:** 2013-03-01

**Authors:** Adam J. Carroll

**Affiliations:** ^1^Australian Research Council Centre of Excellence in Plant Energy Biology, Australian National UniversityCanberra, ACT, Australia

**Keywords:** *Arabidopsis*, sub-cellular proteomics, proteomics, ribosomes, cytosolic ribosomes, plants, translation, 80S ribosomes

## Abstract

The cytosolic ribosomal proteome of *Arabidopsis thaliana* has been studied intensively by a range of proteomics approaches and is now one of the most well characterized eukaryotic ribosomal proteomes. Plant cytosolic ribosomes are distinguished from other eukaryotic ribosomes by unique proteins, unique post-translational modifications and an abundance of ribosomal proteins for which multiple divergent paralogs are expressed and incorporated. Study of the *A. thaliana* ribosome has now progressed well beyond a simple cataloging of protein parts and is focused strongly on elucidating the functions of specific ribosomal proteins, their paralogous isoforms and covalent modifications. This review summarises current knowledge concerning the *Arabidopsis* cytosolic ribosomal proteome and highlights potentially fruitful areas of future research in this fast moving and important area.

## Ribosomes – A Fundamentally Important Target for Basic and Applied Science

Ribosomes – the ribonucleoprotein complexes responsible for catalyzing translation – the mRNA-guided synthesis of proteins from aminoacyl-tRNA, GTP and ATP substrates – have fascinated biologists since their Nobel Prize-winning discovery by George E. Palade in 1955 (Zorca and Zorca, 2011). Understanding how ribosomes are made by cells (ribosome biogenesis and structure), how they work (ribosome molecular mechanics) and how they are controlled through transcriptional, translational, and post-translational mechanisms is of fundamental importance for several reasons. The most obvious reason relates to the fundamental role of ribosomes in the generation of proteomes. Like DNA replication and transcription, translation is a basic requirement for life and an integral component of the Central Dogma of molecular biology.

Understanding the molecular mechanics of different ribosomes will increase our capacity to: (1) design bioactive agents to alter their function (Kannan et al., 2012) and (2) rationally engineer them to modify their performance (Piekna-Przybylska et al., 2008; Santoro et al., 2009) or even provide them with completely new functions – e.g., the residue-specific incorporation of unnatural amino acids into designer polypeptides with novel research and industrial applications (Bain et al., 1992; Benner, 1994; Taira et al., 2005; Neumann et al., 2010; Neumann, 2012). Custom-engineered ribosomes have even been used to create synthetic Boolean information processing networks that control gene expression according to rationally designed logic (Rackham and Chin, 2005, 2006). Despite these numerous examples illustrating the power of ribosome engineering in non-plant species, there are, to the author’s knowledge, no published examples of applied ribosome engineering in plants. It seems inevitable that powerful applications of plant ribosome engineering will emerge in time.

Another reason for the fundamental importance of ribosome research relates to chemical and energy resource usage. In rapidly dividing yeast cells, up to at least 60% of transcriptional activity is devoted to ribosome biogenesis alone, consuming vast amounts of nitrogen (N), phosphorous (P), and energy while translation itself represents a further major demand on N and energy reserves (Warner, 1999; Piques et al., 2009). Understanding the mechanisms controlling ribosome biogenesis and translation in plants could therefore have profound implications for the management, engineering, and utilization of the enormous chemical energy fluxes in natural and agricultural ecosystems.

## Eukaryotic Ribosomes – More Complex Machines to Build More Complex Organisms

The gross structure of ribosomes is essentially the same between prokaryotes and eukaryotes in that they are both comprised of ribosomal RNAs (rRNAs) and proteins (r-proteins) in large and small subunits. However, the ribosomes of eukaryotes exhibit greater structural complexity, reflecting the greater complexity of molecular mechanics observed in eukaryotic translation (Kapp and Lorsch, 2004). In eukaryotes, nuclear-encoded proteins (i.e., the vast majority of proteins) are synthesized on 80S cytosolic ribosomes which are distinguished from the 70S prokaryotic-type ribosomes of bacteria, mitochondria, and plastids by their larger size and higher number of proteins (∼80 versus ∼54). Each 80S eukaryotic ribosome is comprised of a large 60S subunit (50S in prokaryotic ribosomes) containing three rRNA molecules (5S, 5.8S, and a 23S-like rRNA ranging between 25S and 28S in plants) and up to 47 different r-proteins and a small 40S subunit (30S in prokaryotic ribosomes) containing a single 18S rRNA and up to 33 different r-proteins (Wilson and Doudna Cate, 2012).

The precise reasons for all the specific differences between prokaryotic and eukaryotic translation machineries and processes remain largely unknown. However, it seems likely that the higher complexity of eukaryotic translation evolved in response to the following needs which, intuitively, seem likely to be characteristic of eukaryotic organisms: (1) to translate with a greater priority toward fidelity and control over speed of ribosome biogenesis; (2) to efficiently and accurately translate mRNAs having (and encoding proteins having) a wider range of primary and secondary structures; (3) to have greater control over the relative rates of translation of specific mRNAs; (4) to have a greater capacity for spatiotemporal ribosome heterogeneity in order to tailor the translation process for different subcellular locations, cell types, and developmental stages (Giavalisco et al., 2005; Komili et al., 2007; Sugihara et al., 2010; Xue and Barna, 2012).

Among eukaryotes, the cytosolic ribosomes of yeast (*Saccharomyces cerevisiae*), rat (*Rattus norvegicus*), human (*Homo sapiens*), and *Arabidopsis* (*Arabidopsis thaliana*) have been the most extensively characterized. Primarily through the extensive r-protein sequencing and gene cloning efforts of Wool et al. (1995), rat liver ribosomes were the first eukaryotic ribosomes for which a presumed complete list of r-proteins became available and has since served as a useful model for r-protein nomenclature in yeast (Mager et al., 1997) and plants (Barakat et al., 2001) although some inconsistencies do still exist between the r-protein nomenclatures of yeast and other eukaryotes. Efforts to characterize the cytosolic ribosomes of other eukaryote lineages have since revealed that all 79 of the r-protein families present in mammalian ribosomes are also represented in the ribosomes of yeast and plants (Wilson and Doudna Cate, 2012) although an additional plant-specific r-protein family known as acidic stalk protein P3 has been identified in the ribosomes of plants (Szick et al., 1998; Barakat et al., 2001; Chang et al., 2005; Carroll et al., 2008). This deep conservation of the protein composition of eukaryotic ribosomes suggests that the archetypal eukaryotic ribosome evolved very early in eukaryote evolution and that all of the r-protein families are important for ribosome function. That said, considerable primary sequence divergence has occurred between r-protein orthologs of different eukaryote lineages (Wool et al., 1995) and between r-protein paralogs within individual species that have emerged through gene duplication events during eukaryote evolution (Barakat et al., 2001). Hence, a major current focus of ribosome-related research is to elucidate the adaptive and physiological significance of these divergences and the ribosome heterogeneity that they enable (Wool et al., 1995; Wilson and Doudna Cate, 2012; Xue and Barna, 2012).

## Plant Ribosomes – A Challenging Target for Proteomics

Plants offer unique technical challenges to researchers of ribosomal proteomes. Firstly, in addition to the cytosolic and mitochondrial ribosomes found in mammals and fungi, plants contain a third type of ribosome in the plastid thus introducing more potential for cross-contamination of ribosome preparations and more potential for ambiguity with respect to the localization of r-proteins when they are detected in multiple cellular fractions. Protocols for the isolation of cytosolic ribosomes from plants must therefore incorporate special measures to avoid contamination from organelle ribosomes.

Another challenge associated with the study of plant ribosomes is that the possible degree of heterogeneity is particularly high (Giavalisco et al., 2005). While mammalian r-proteins are usually represented by only a single expressed gene (Sugihara et al., 2010) and yeast r-proteins are each represented by only one or two, often encoding identical proteins (McIntosh and Warner, 2007), the situation is far more complex in higher plants. Indeed, high heterogeneity appears to be particularly characteristic of higher plants with much less paralog heterogeneity being observed in 80S ribosomes of the green alga, *Chlamydomonas reinhardtii* (Manuell et al., 2005). A survey of the *Arabidopsis* genome (Barakat et al., 2001) revealed that none of the 80 different r-protein families were encoded by a single-copy gene. Rather, most were found to be encoded by three or four transcribed genes. These paralogs could theoretically combine to form more than 10^34^ different ribosomes, not including different post-translational modifications (PTMs; Hummel et al., 2012). This striking potential for heterogeneity is likely attributable to the sessile nature of plants and their greater need to be adaptable under changing environments than animals, who have more capacity to avoid environmental fluctuations.

A major ongoing challenge has been to determine not only which r-protein families but precisely which of the 251 r-protein genes encoded by the *A. thaliana* genome (Barakat et al., 2001; Chang et al., 2005) are transcribed and translated into proteins that are incorporated into ribosomes. As will be discussed shortly, a collection of proteomic studies (see Table 1) have confirmed the presence of all but one of the 81 predicted r-protein families and, in the case of many families, the presence of multiple distinct paralogous family members in *Arabidopsis* ribosomes (Chang et al., 2005; Giavalisco et al., 2005; Carroll et al., 2008; Piques et al., 2009; Turkina et al., 2011; Hummel et al., 2012).

**Table 1:** **Proteomic studies of *Arabidopsis thaliana* ribosomes – an overview**.

Study	Tissue	Methods	Detected proteins/information provided
Giavalisco et al. (2005)	Leaves	Differential centrifugation and sucrose gradients, 2D-E, and MALDI-TOF PMF	Representatives of 60 r-protein families including 87 distinct r-protein identifications based mainly on intact peptide masses
Chang et al. (2005)	Cell suspension	Detergent extraction, ultracentrifugation and sucrose gradient followed by 2D-E, MALDI-TOF PMF, and some LC-MS/MS. Barium hydroxide treatment of phospho-proteins	Representatives of 74 r-protein families including 77 family member-specific claims based mainly on intact peptide masses
Carroll et al. (2008)	Cell suspension	Differential centrifugation and double sucrose cushion purification followed by 1D-SDS-PAGE and LC-MS/MS. Titanium dioxide phosphopeptide enrichment. Use of pepsin and chymotrypsin in addition to trypsin to increase coverage of low MW r-proteins. Bioinformatic analysis to quantify gene family member-specific MS/MS evidence	Representatives of 80 r-protein families including 87 family member-specific identifications based on detailed MS/MS analysis. 30 residue-specific post-translational modification sites including Initiator methionine removal, N-terminal acetylation, serine phosphorylation, lysine mono and tri-methylation, and N-terminal proline dimethylation
Piques et al. (2009)	Leaves sampled at different times of the diurnal cycle	Polysome fractions were isolated by detergent extraction and sucrose gradient fractionation as described in Kawaguchi et al. (2003). Proteins were then acetone-precipitated, trypsin digested, and analyses directly by nano-LC-MS/MS with data-dependent MS/MS acquisition. Absolute protein concentrations were estimated using the emPAI scoring method of Ishihama et al. (2005)	Estimates of absolute (mol%) concentrations of r-proteins in different polysome fractions at different times of day and night. MS/MS evidence provided to support the detection of representatives from 71 r-protein families. MS/MS evidence for proteotypic peptides from 92 specific r-proteins collectively across the various polysome samples
Turkina et al. (2011)	Leaves sampled at day and night	Detergent extraction followed by ultracentrifugation through sucrose cushion and then nano-LC-MS/MS for qualitative analysis. Also, quantitative phosphoproteomic analysis using differential isotopic labeling of tryptic peptides by methyl esterification with IMAC enrichment of phosphopeptides prior to LC-MS/MS. Provide counts of family member-specific peptides	Representatives from 72 r-protein families including family member-specific identifications of 71 r-proteins based on MS/MS. New phosphorylation sites on S6, S2 and L29. Diurnal changes in phosphorylation levels of S6 and L29
Hummel et al. (2012)	Leaves ± sucrose feeding	Immunopurification of ribosomes from a transgenic *A. thaliana* line expressing a His FLAG Tagged form of RPL18B (Zanetti et al., 2005; Mustroph et al., 2009). Relative quantitation with simultaneous identification by label-free LC-MS^E^ analysis	Representatives of 74 r-protein families including 166 family member-specific identifications. Changes in the levels of RPS3aA, RPS5A, RPL8B, and RACK1 in response to sucrose feeding were particularly evident

*Brief details about each of the major proteomic studies of *A. thaliana* ribosomes are provided. PMF, Peptide Mass Fingerprinting; 2D-E, Two-dimensional gel electrophoresis; IMAC, Immobilized Metal Affinity Chromatography; LC-MS/MS, Liquid Chromatography – Tandem Mass Spectrometry; MALDI-TOF, Matrix Assisted Laser Desorption Ionization Time-of-Flight Mass Spectrometry*.

Determining precisely which members of r-protein families are incorporated into ribosomes depends on the ability to confidently discriminate between those protein isoforms. In theory, proteotypic peptides – peptides specific to a single gene-product – may be generated from trypsin digestion of most r-proteins and confident detection of these may be used as evidence for the presence of their corresponding specific gene products. However, *in silico* analysis has revealed that 10 r-protein families (S18, S29, S30, L11, L21, L23, L36a, L38, L40, and L41) exhibit no sequence divergence within them while others (S15a, S16, S2, S20, S4, L11, L35a, L39, and L9) include some members predicted to generate proteotypic peptides and others that would not (Carroll et al., 2008). Hence, proteomics alone will be of limited use for assessing the heterogeneity of the ribosomal proteome in these perfectly homologous r-proteins. This is non-trivial given intriguing observations that, in yeast, independent deletion of paralogous genes encoding sequence-identical proteins cause readily distinguishable phenotypes, suggesting that these paralogous genes are functionally non-equivalent despite the fact that the proteins they encode are predicted to have identical amino acid sequences (Komili et al., 2007; McIntosh and Warner, 2007). Promoter analysis using reporter gene constructs expressed under the promoters of different r-protein paralogs, as exemplified in the L16 family (Williams and Sussex, 1995), will continue to be valuable in determining the physiological significance of these different paralogs.

## Defining the *Arabidopsis* Cytosolic Ribosomal Proteome – Progress to Date

Several proteomic studies of *Arabidopsis* cytosolic ribosomes have been reported in the literature – each employing its own unique combination of methods for purification, gel separation, mass-spectrometric detection and data analysis (summarized in Table 1). In the earliest of these reports, Giavalisco et al. (2005) combined differential centrifugation and sucrose density gradient purification of *Arabidopsis* leaf ribosomes with 2D-gel electrophoresis and Matrix Assisted Laser Desorption Ionization Time-of-Flight Mass Spectrometry (MALDI-TOF-MS)-based Peptide Mass Fingerprinting (PMF) to identify protein spots corresponding to 87 distinct r-protein gene products representing 60 of the 80 r-protein families. The authors highlighted low molecular weights, high pIs and low numbers of tryptic cleavage sites as possible reasons for the non-detection of the other 20 predicted families of r-proteins in their study. A key finding of the study was that at least 21 of the 60 detected r-protein families were represented by two or more distinct r-proteins (distinct AGIs) and that >45% of the distinct r-proteins detected were represented by 2–13 separate spots. Indeed, this confirmed earlier predictions by Barakat et al. (2001) of high ribosomal heterogeneity in plants due to the frequent expression of multiple divergent paralogous r-protein genes. However, it also suggested that plant r-proteins exist in a much wider variety of modification states than those of other organisms (Giavalisco et al., 2005).

Shortly after Giavalisco et al. (2005) published their study of *Arabidopsis* leaf ribosomes, Chang et al. (2005) published an independent proteomic survey of 80S ribosomes isolated from heterotrophic *Arabidopsis* cell suspensions. This study combined detergent-based tissue lysis with differential centrifugation, sucrose gradient purification, 1D- and 2D-gel electrophoresis with MALDI-TOF-MS PMF and in some cases Liquid Chromatography – Electrospray Ionization – Quadrupole – Time-of-Flight – Tandem Mass Spectrometry (LC-ESI-Q-TOF-MS/MS). Protein assignments based on ∼850 peptide identifications (mostly based on MALDI-TOF-MS with 172 based on MS/MS) provided evidence for the ribosomal incorporation of 14 previously undetected r-protein families, bringing the total number of detected families to 74 and leaving only six undetected. This study also presented new evidence to support the identification of particular r-protein family members by reporting the masses of ions assigned to tryptic peptides predicted to belong to only one specific gene product. On this basis, Chang et al. (2005) provided paralog-specific evidence for 77 r-proteins with the following 25 r-protein families being represented in the cytosolic ribosomal proteome by more than one structurally distinct member: S3a, S6, S7, S10, S12, S14, S15, S15a, S16, S19, S23, S24, Sa, P0, P2, L4, L7, L7a, L8, L10, L10a, L18a, L26, L27, L31.

Even after considerable efforts by Giavalisco et al. (2005) and Chang et al. (2005) to define the *Arabidopsis* cytosolic ribosomal proteome, clear opportunities to gain further insight remained. *In silico* analyses suggested that many r-protein families for which gene-specific peptides were predicted to exist had still not been resolved, suggesting that a higher-coverage proteomic analysis based on peptide MS/MS rather than PMF might yield further evidence with which to resolve particular paralogs. Moreover, while limited tissue-type sampling was one possible reason for the non-detection of some r-proteins, *in silico* analyses also suggested that the six small and basic r-protein families remaining undetected might have been missed by the previous studies because their tryptic peptides were very small and that their detection may have been aided through the use of complementary proteases yielding larger fragments (Carroll et al., 2008).

Prompted by the above observations, Carroll et al. (2008) undertook a systematic analysis of highly pure 80S ribosomes isolated from *Arabidopsis* cell suspensions by combining an optimized ribosome isolation procedure with 1D gel electrophoresis, LC-ESI-Q-TOF-MS/MS analysis of excised gel bands (using three different proteases on low MW bands to capture larger peptides) and a custom data analysis pipeline to provide deep proteome coverage and high-confidence paralog-specific identifications. This analysis, based on 1446 high-quality MS/MS spectra matching to 795 peptide sequences, provided high-confidence evidence for the presence of 79 of the 80 predicted r-protein families in the ribosomes of *Arabidopsis*, including five previously undetected r-protein families: S29, S30, L29, L36a, and L39.

To date, the only predicted r-protein family yet to be detected in *Arabidopsis* ribosomes is the extremely basic (predicted pI of 13.4) and small (3.5 kDa) L41. The four paralogous L41 genes in the *A. thaliana* genome (At2g40205, At3g08520, At3g11120, and At3g56020) encode identical proteins with the amino acid sequence MRAKWKKKRMRRLKRKRRKMRQRSK. The strong conservation between eukaryotes of genes encoding this putative r-protein suggests that it is most likely a component of *Arabidopsis* ribosomes. X-ray crystallography has shown that the yeast ortholog of *Arabidopsis* L41 forms a bridge between the 40S and 60S subunits (Wilson and Doudna Cate, 2012), deep in the ribosome. For this reason, its non-detection so far in *Arabidopsis* ribosomes seems more likely to be due to technical limitations of the LC and MS detection approaches used rather than its absence in samples. Given that trypsin is not expected to yield useful peptides from L41, its detection in ribosomes is likely to require either targeted top-down LC-MS methods (Odintsova et al., 2003) or x-ray crystallography. Top-down LC-MS analyses are likely to require special chromatographic conditions as, with a predicted pI of 13.4, L41 is likely to be highly charged and therefore unlikely to be retained under typical reverse-phase pH conditions used in non-targeted top-down proteomics. Perhaps synthetic L41 peptides will prove useful as positive controls for method development and validation.

Carroll et al. (2008) provided strong MS/MS evidence to support the identification of 87 specific r-protein paralogs in total, including 32 not previously reported by Chang et al. (2005). These paralog-specific identifications confirmed previous reports of heterogeneity within S10, S12, S14, S15, S19, S24, S3a, S6, S7, Sa, P0, P1, P2, L10, L10a, L18a, L26, L27, L4, L7, L7a, and L8 (Chang et al., 2005) and provided strong evidence for previously unreported heterogeneity within a further 19 families, namely: S11, S2, S21, S25, S27a, S3, P1, L13a, L17, L18, L22, L23a, L28, L32, L35, L36, L37a, L5, and L6. In the case of six families – namely S15a, S16, S23, L19, and L31 – the paralog-specific detection of only a single family member contrasted with reports of Chang et al. (2005) of heterogeneity within those families. While the fact that Carroll et al. (2008) used much higher-stringency filters for their paralog-specific identifications should be considered when comparing these datasets, it is possible that these discrepancies were due at least in part to the tendencies of MALDI-TOF-MS and LC-ESI-Q-TOF-MS/MS to preferentially ionize different peptides (Stapels and Barofsky, 2004). Hence, each platform may have preferentially detected paralog-specific peptides from different r-proteins. Other possible contributors to differences in detected r-protein profiles are, of course, differences in tissue types and growth conditions. Given these considerations, the limited range of analytical techniques employed to date and the fact that new r-protein PTMs have only recently been detected (Turkina et al., 2011), it seems likely that the true extent of ribosome heterogeneity is greater than indicated by any individual study or, indeed, all the studies collectively. For the reader’s convenience, Table S1 in Supplementary Material aligns and summarizes the r-protein identifications and post-translational modification detections reported to date across all of the major proteomic analyses of *A. thaliana* ribosomes (listed in Table 1).

## Type II S15a Proteins: Components or Contaminants of the *Arabidopsis* Cytosolic Ribosome?

The *Arabidopsis* genome encodes for two evolutionarily distinct classes of S15a r-protein, commonly denoted type I and type II (Chang et al., 2005). There is strong evidence that the type II forms obtained functional mitochondrial targeting sequences to become part of the mitochondrial ribosome during the evolution of higher plants (Adams et al., 2002). Indeed, Carroll et al. (2008) detected paralog-specific peptides for both type II S15a proteins (S15aB and S15aE) in *Arabidopsis* mitochondrial ribosome preparations.

The detection of type II S15a sequences in their crude ribosomal pellet led Chang et al. (2005) to hypothesize that type II S15a proteins might be part of the cytosolic ribosome. However, an alternative explanation for this observation lies in the use of four membrane-solubilizing detergents (1% each of Triton X-100, Brij 35, Tween-40, and NP-40) – which would have dissolved mitochondrial membranes (Gurtubay et al., 1980) and released mitochondrial ribosomes and other mitochondrial proteins prior to pelleting of ribosomes by ultracentrifugation – in the ribosome extraction buffers of Chang et al. (2005). Hence, although other mitochondrial ribosomal proteins were not detected, the crude ribosome pellet of Chang et al. (2005) in which the type II proteins were detected (they were not reported in the sucrose gradient purified ribosomes) most probably contained at least a considerable portion of the mitochondrial ribosome population of their experimental cells-albeit at inherently low molar % levels reflecting their low cellular abundance relative to cytosolic ribosomes (Piques et al., 2009). A mitochondrial origin of the type II S15a proteins cannot, therefore, be ruled out on the basis of that analysis. In contrast, Carroll et al. (2008), who used a detergent-free ribosome extraction buffer containing 0.45 M mannitol as osmoticum to prevent osmotic bursting of organelles and subjected their tissue homogenates to 1500 × *g* × 5 min, 16,000 × *g* × 15 min, and 30,000 × *g* × 30 min centrifugation steps to remove nuclei/chloroplasts, mitochondria and large aggregates of poorly defined insoluble materials prior to ultracentrifugation, did not observe a single peptide mapping to type II S15a proteins in their 80S ribosome preparations despite finding strong MS/MS evidence for proteotypic peptides from type I S15a proteins.

Methods are available to resolve 80S and 70S chloroplast ribosomes (Yamaguchi, 2011). However, given that the 70S and 80S ribosomes of *C. reinhardtii* sedimented closely on sucrose gradients (Yamaguchi et al., 2003) and that mitochondrial ribosomes from higher plants have been observed to sediment anywhere between 70S (Vasconcelos and Bogorad, 1971; Pinel et al., 1986) and 78S (Leaver and Harmey, 1973, 1976; Pring, 1974), the above observations highlight the importance of early fractionation steps, orthogonal to sucrose gradient purification, in obtaining pure cytosolic ribosomes required for confident discrimination of cytosolic and organellar ribosomal proteomes. In this author’s view, this technical point is worth highlighting given the potential functional and evolutionary significance of parallel-targeting of r-proteins to multiple ribosomes in eukaryotic cells and the fact that just a few simple protocol modifications could greatly enhance the utility of future studies in addressing this important possibility.

## “Non-Ribosomal” Ribosome-Associated Proteins

### What is a non-ribosomal protein?

Each of the major efforts to qualitatively define the *Arabidopsis* ribosomal proteome has reported the detection of “non-ribosomal” proteins in purified ribosomes (Chang et al., 2005; Giavalisco et al., 2005; Carroll et al., 2008; Hummel et al., 2012). However, the reporting of “non-ribosomal” proteins in purified ribosomes begs the question “how do we define ribosomal proteins?” As many new proteins not orthologous to the original set of 79 proteins originally labeled as core r-proteins by Wool et al. (1995) continue to be confidently detected in purified ribosome samples (Hummel et al., 2012), the classic view of ribosomes as a well-defined proteomic entity with a consistent stoichiometry is rapidly giving way to an increasingly fuzzy model of the ribosomal proteome in which a well-defined set core r-proteins (some of which may not always be associated with ribosomes) serve as a docking station for a poorly defined set of ribosome-associated regulatory proteins for which the natures and functions of their ribosome interactions are unclear (Gilbert, 2011; Xue and Barna, 2012). Due to the large number of ribosome-associated proteins that have now been reported and the fact that further experiments will be required to determine which associations represent *bona-fide* interactions as opposed to non-specific binding, an exhaustive list will not be provided here. Rather, the following sections highlight and discuss some examples for which *bona-fide* functions are either well established or worthy of further investigation based on independent information (which will be explained below).

### RACK1 and eIF6

Of all the ribosome-associated proteins detected so far in *A. thaliana* ribosomes, orthologs of the mammalian Receptor of Activated C Kinase (RACK1) are the most consistently detected. The RACK1A protein encoded by At1g18080 has been reported in all major proteomic surveys of *A. thaliana* ribosomes to date (Chang et al., 2005; Giavalisco et al., 2005; Carroll et al., 2008; Hummel et al., 2012). A second RACK1 ortholog (RACK1B) encoded by At1g48630 has also been detected in three independent studies (Chang et al., 2005; Carroll et al., 2008; Hummel et al., 2012) but RACK1C (At3g18130), the third of the three known RACK1 genes in the *A. thaliana* genome has not yet been detected in *A. thaliana* ribosomes. The close association of RACK1 with mammalian and yeast ribosomes has been known for some time and its role as a key regulatory component of the eukaryotic translation machinery is now well appreciated (Jakob et al., 2004). While RACK1 does not appear to be essential for translation in yeast, its absence decreases the efficiency of translation and steady state levels of numerous proteins (Shor et al., 2003). RACK1 is believed to play a key role in 80S ribosome assembly by directing the phosphorylation (by activated C Kinase) and release of eukaryotic Translation Initiation Factor 6 (eIF6) from the 60S subunit, thus allowing assembly of the 80S ribosome (Ceci et al., 2003; Guo et al., 2011).

In *A. thaliana*, a collection of studies have identified RACK1 as a key integrator and mediator of hormonal control over translation (Chen et al., 2006; Guo and Chen, 2008; Guo et al., 2009a,b, 2011). In particular, evidence suggests that abscisic acid (ABA) may down-regulate translation generally by inhibiting the transcriptional expression of RACK1 and eIF6 mRNAs, although the mechanism by which ABA controls the levels of these mRNAs is currently unclear (Guo et al., 2011). Interestingly, the amount of RACK1A (but not RACK1B) associated with ribosomes/polysomes increased significantly in response to sucrose feeding in *A. thaliana* (Hummel et al., 2012) – a response that is known to involve ABA signaling pathways (Laby et al., 2000). The *Arabidopsis* genome encodes two homologs of eIF6 – At3g55620 (eIF6A) and At2g39820 (eIF6B). While both proteins have been demonstrated to interact physically with RACK1 (Guo et al., 2011), only the eIF6A protein has been detected in the ribosomes of *A. thaliana* leaf and suspension cells (Carroll et al., 2008; Hummel et al., 2012). This is consistent with mRNA expression patterns which indicate that while eIF6A mRNA is expressed ubiquitously, eIF6B mRNA is mainly expressed in flower buds, stamens, and pollen (Guo et al., 2011). It remains to be seen whether eIF6B is present in the ribosomes of these tissues.

### 20S proteasome

The 20S proteasome forms part of the 26S proteasome complex responsible for the proteolysis of many proteins (particularly those carrying poly ubiquitin tails) in eukaryotic cells (Yang et al., 2004). Subunits of the 20S proteasome were detected in polysomal bands on sucrose density gradients by Chang et al. (2005) and also in crude ribosomal pellet by Giavalisco et al. (2005) but not in the highly purified ribosome samples of Carroll et al. (2008). Because of the high abundance and similar sedimentation coefficient of the proteasome complex (when associated with other complexes), there has been some uncertainty whether the association of the proteasome with ribosomes was due to a *bona-fide* *in vivo* interaction or simply a non-specific interaction between abundant complexes or simple co-sedimentation (Chang et al., 2005). Indeed, the fact that proteasome subunits were not reported in epitope-tag purified *A. thaliana* ribosomes in the same manner as RACK1 (Chang, 2006) suggests that if a *bona-fide* interaction between the proteasome complex and *A. thaliana* ribosomes exists, it is more labile than the interaction between ribosomes and RACK1. That said, given that the proteasome is thought to play a role in degrading defective ribosomal products (proteins that result from errors in translation or folding) representing some 30% of newly synthesized proteins, it would seem efficient to have proteasome complexes localized at the point of protein synthesis to prevent the escape of potentially toxic defective proteins into the cytoplasm. Another possible explanation may lie in the major role played by the proteasome in ribosome biogenesis (Stavreva et al., 2006).

### Ferritin

Only four ribosome-associated proteins were detected in the ribosome preparations of Carroll et al. (2008). Three of these – namely RACK1A, RACK1B, and eIF6 – have been discussed above. The fourth ribosome-associated protein detected was identified as FERRITIN 3 (FER3; At3g56090). The fact that so few ribosome-associated proteins were detected in these ribosomes and the striking absence of obvious abundant non-specific binding proteins suggests that FER3 was indeed tightly associated with these ribosomes. The FER3 protein has also been detected in small polysome fractions isolated from the leaves of *A. thaliana* plants in the dark (Piques et al., 2009). This is intriguing given that FER3 is a nuclear-encoded chloroplast-targeted protein that, according to the SUBA database (Heazlewood et al., 2007), has been repeatedly detected in chloroplast preparations by mass spectrometry. In humans, ferritin has been demonstrated to regulate folate metabolism by controlling the translation of cytosolic serine hydroxymethyltransferase (cSHMT) via binding to ferritin-responsive internal ribosome entry site (IRES) in the 5′UTR of the cSHMT mRNA (Woeller et al., 2007). The H ferritin involved was also shown to interact physically with the mRNA-binding protein CUGBP1 which is known to interact with the α and β subunits of eukaryotic translation initiation factor 2 (eIF2; Woeller et al., 2007). Together, the above observations suggest that the existence of similar mechanisms involving FER3 in *A. thaliana* should be investigated. The link with chloroplasts is particularly intriguing since it is possible to imagine a mechanism whereby FER3 mediates coordination between the translational activity of cytosolic ribosomes and the function of chloroplasts in response to iron-based signals.

## Post-Translational Modifications and the Need for “Top-Down” Approaches

Eukaryotic ribosomes are well-known to be rich in many kinds of PTMs. The diversity and conservation of PTMs of r-proteins observed across different eukaryote lineages has been reviewed elsewhere (Carroll et al., 2008) and will not be covered again here. Instead, the discussion of PTMs in this review will focus on providing an updated overview of current knowledge concerning PTMs of *A. thaliana* r-proteins. The different types of PTMs detected in *A. thaliana* cytosolic ribosomes include initiator methionine removal, N-terminal acetylation, serine phosphorylation, lysine mono-, and tri-methylation, and N-terminal proline dimethylation (Chang et al., 2005; Carroll et al., 2008; Turkina et al., 2011). Specific PTM reports are listed in Table 2. Some particularly important issues concerning *A. thaliana* r-protein PTMs are discussed below.

**Table 2:** **Post-translational modifications reported to date in *A. thaliana* ribosomal proteins**.

Family	Loci	Post-translational modifications	Reference
L10a	At1g08360 (L10aA)	^90^K_m3_	a
L10a	At2g27530 (L10aB)	^90^K_m3_	a
L10a	At1g08360 (L10aA), At2g27530 (L10aB), At5g22440 (L10aC)	−Met, ^N-term^S_Ac_	a
L12	At2g37190 (L12A), At3g53430 (L12B), At5g60670 (L12C)	−Met, ^N-term^P_m2_, ^3^K_m3_	a
L12	At2g37190 (L12A), At3g53430 (L12B), At5g60670 (L12C)	−Met, ^N-term^P_Ac_	b
L13	At3g49010 (L13B)	^137^S_phospho_	a
L15	At4g16720 (L15A)	^N-term^G_Ac_	b
L18	At3g05590 (L18B), At5g27850 (L18C)	−Met	a
L21	At1g09590 (L21A), At1g09690 (L21C), At1g57660 (L21E), At1g57860 (L21G)	−Met	a
L28	At2g19730 (L28A), At4g29410 (L28C)	^N-term^A_Ac_	a,b
L28	At4g29410 (L28C)	^N-term^A_Ac_	a
L29	At3g06700 (L29A)	^58^S_phospho_	c
L32	At4g18100 (L32A), At5g46430 (L32B)	−Met	a
L36	At5g02450 (L36C)	−Met	a
L36a	At3g23390 (L36aA), At4g14320 (L36aB)	^55^K_m1_	a
P0	At3g09200 (P0B)	^305^S_phospho_	c
P0	At3g11250 (P0C)	^305^S_phospho_	a
P1	At1g01100 (P1A), At4g00810 (P1B), At5g47700 (P1C)	^102/103^S_phospho_	a,c
P2	At2g27720 (P2A), At2g27710 (P2B), At3g44590 (P2D)	^105^S_phospho_	a
P3	At4g25890 (P3A), At5g57290 (P3B)	^107^S_phospho_	a
S2	At2g41840 (S2C)	^273^S_phospho_	c
S3	At2g31610 (S3A), At5g35530 (S3C)	−Met, ^N-term^A_Ac_	a
S5	At2g37270 (S5A)	−Met, ^N-term^A_Ac_	a,b
S5	At3g11940 (S5B)	−Met, ^N-term^A_Ac_	a
S6	At4g31700 (S6A)	^240^S_phospho_	a,b,c
S6	At5g10360 (S6B)	^240^S_phospho_	a,c
S6	At4g31700 (S6A), At5g10360 (S6B)	Mono-, di-, tri-, and tetra-phospho at unknown sites in C terminal region	b
S6	At4g31700 (S6A), At5g10360 (S6B)	^231^S_phospho_	c
S7	At1g48830 (S7A)	^N-term^M_Ac_	a
S15	At1g04270 (S15A), At5g09510 (S15D)	−Met, ^N-term^A_Ac_	a,b
S16	At2g09990 (S16A), At5g18380 (S16C)	−Met, ^N-term^A_Ac_	b
S18	At1g22780 (S18A), At1g34030 (S18B), At4g09800 (S18C)	−Met, ^N-term^S_Ac_	a,b
S20	At3g45030 (S20A), At5g62300 (S20C)	−Met, ^N-term^A_Ac_	a
S20	At3g47370 (S20B)	−Met, ^N-term^A_Ac_	a,b
S21	At3g53890 (S21B)	^N-term^M_Ac_	a,b
S27	At2g45710 (S27A), At3g61110 (S27B)	−Met	a
Sa	At1g72370 (SaA)	−Met, ^N-term^A_Ac_	a

*Reports of post-translational modifications of *A. thaliana* r-proteins are listed in order of r-protein family. Each row corresponds to an individual report of a particular modified peptide. The paralog(s) to which each peptide may be mapped theoretically is indicated in column “Loci” with the listing of several paralogs indicating ambiguity with respect to which paralog carried the indicated modification and the listing of a single paralog indicating that the detected modified peptide was specific to a single paralog. In the column “Post-translational modifications,” “–Met” indicates removal of the initiator methionine while other modification(s) reported to be associated with the peptide are indicated in the format *^residue position^X_modification type_* where *X* is the one-letter code of the modified amino acid residue. All residue positions are given with the initiator methionine as position 1 regardless of whether this methionine is removed. Abbreviations used include: N-term, N terminus; Ac, Acetyl; phospho, phosphorylation. Methylation modification types are abbreviated as m*Y* where *Y*, the number of methyl groups added. In the references column, (a) Carroll et al. (2008), (b) Chang et al. (2005), and (c) Turkina et al. (2011)*.

That phosphorylation sites exist on S6 and the acidic stalk P proteins has been well established for some time, primarily from work in *Zea mays* (Szick-Miranda and Bailey-Serres, 2001; Williams et al., 2003). The conservation of these modifications in *A. thaliana* ribosomes has been confirmed more recently (Chang et al., 2005; Carroll et al., 2008; Turkina et al., 2011). However, new phosphorylation sites continue to emerge as new tissues are analyzed and new methods of analysis are employed. For example, Carroll et al. (2008) recently reported a previously undiscovered phosphorylation site on L13(At3g49010). It should be noted that this r-protein is not homologous to the human r-protein L13a which has been shown to act as an mRNA-binding translational suppressor upon being released from human ribosomes by phosphorylation following treatment of cells with interferon-γ (Mazumder et al., 2003). Interestingly, phosphorylated L13 was not detected in a recent quantitative phosphoproteomic analysis of *A. thaliana* leaf cytosolic ribosome despite the detection of previously undetected phosphorylation sites at Ser_231_ of S6 and Ser_58_ of L29A (Turkina et al., 2011). Phosphorylation of the human ortholog of L29 has also been detected (Molina et al., 2007; Wang et al., 2008). Together, these observations highlight the likely plasticity of *A. thaliana* L13 and L29A phosphorylation and suggest that potential roles of L13 and L29 phosphorylation in translational control need targeted research. The extent to which these and other modifications are conserved across different plant species remains to be seen. However, given the divergence of PTMs seen between the major eukaryote lineages (Carroll et al., 2008), the possibility that the gain or loss of specific r-protein PTM sites by different plant species during plant evolution could have played a role in ecological specialization of plants is too tantalizing not be explored.

Another important point relates to the potential role of post-translational modification in ribosome heterogeneity. Giavalisco et al. (2005) suggested that the observation that >45% of specific r-proteins were detected in 2–13 spots was indicative of each being present in various modification states. In contrast, none of the 30 modification sites identified by Carroll et al. (2008) were also detected in an unmodified form. One possible explanation for the greater diversity of modification states observed by Giavalisco et al. (2005) may lay in the higher diversity of cell types expected in leaves compared to relatively homogeneous and undifferentiated cell cultures analyzed by Chang et al. (2005) and Carroll et al. (2008). Another possibility is that the extra spots observed by Giavalisco et al. (2005) were multiply phosphorylated forms that generate multiply phosphorylated peptides that are notoriously difficult to detect directly by mass spectrometry (Choi et al., 2008). Proteolytic degradation, either via natural *in vivo* mechanisms or during analysis, may have also contributed detection of r-proteins across multiple spots (Finnie and Svensson, 2002; Vohradsky et al., 2008).

The application of “top-down” proteomics techniques involving the direct analysis of intact proteins by LC/MS without protease digestion will be invaluable in resolving the issues discussed above (Zhang and Ge, 2011; Zhou et al., 2011). Top-down approaches complement bottom-up approaches by revealing modification states – such as multiple modifications at distal sites or proteolytic protein truncation – that are masked by protease digestion. Similarly, modifications that are difficult to detect in peptide form (e.g., multiply phosphorylated peptides) may be more amenable to detection in the form of modified whole proteins. Top-down approaches have been used extensively to study the composition and PTMs of mammalian (Louie et al., 1996; Odintsova et al., 2003; Yu et al., 2005) and yeast (Arnold et al., 1999; Lee et al., 2002) ribosomes. However, top-down analyses of plant ribosomes have still to be carried out.

A recent quantitative phosphoproteomic analysis has confirmed that phosphorylation not only contributes to cytosolic ribosome heterogeneity in *Arabidopsis*, but the relative abundance of different phosphorylated forms of S6 and L29 change during the diurnal cycle (Turkina et al., 2011). The levels of mRNA transcripts encoding the phosphorylated acidic stalk P proteins P1, P2A, P2B, and P3 have been shown to be highly variable across different organs and tissues in *Zea mays* (Szick-Miranda and Bailey-Serres, 2001). Importantly, while levels of P1, P2A, and P2B (but not P3) proteins in ribosomal extracts were also shown to be variable across different organs and tissues, these levels were poorly correlated with the observed variation in mRNA levels, clearly demonstrating the importance of proteome level studies. This study also demonstrated that the phosphorylation levels of the P1, P2A, and P3 proteins of root tip ribosomes decreased under anoxic conditions.

## The Enzymes that Modify *Arabidopsis* r-Proteins are Largely Unknown

The identification of enzymes responsible for the post-translation modification of ribosomal proteins has progressed much more slowly in *Arabidopsis* than in other eukaryotic systems. While the kinase responsible for *Arabidopsis* S6 phosphorylation has been known for some time (Mizoguchi et al., 1995; Mahfouz et al., 2006), little is known about the enzymes responsible for other modifications of *A. thaliana* r-proteins. In contrast, a variety of *N*-methyltransferases and acetyltransferases responsible for the modification of r-proteins have been identified in yeast and humans (Arnold et al., 1999; Bachand and Silver, 2004; Porras-Yakushi et al., 2005, 2008; Ren et al., 2010; Webb et al., 2010a,b, 2011; Forte et al., 2011). Proteomic analysis of ribosomes isolated from *A. thaliana* mutants perturbed in orthologous or homologous candidate genes encoding potential ribosome-modifying enzymes will almost certainly be a fruitful line of research.

## The Transition from Qualitative to Quantitative Ribosomal Proteomics

With an abundance of qualitative proteomics data suggesting the extreme heterogeneity of cytosolic ribosome populations from whole plant tissues, ribosomal proteomics is now heavily focused on understanding the spatiotemporal distribution and physiological function of this heterogeneity. Ribosome heterogeneity could and probably does occur at many different spatiotemporal scales – from slow developmental changes or constitutive differences in the ribosome populations of distinct organs to rapid changes in minor subcellular populations of ribosomes within single cells. Hence, a major task that will be important for reverse-engineering the physiological function of ribosome heterogeneity will be the use of quantitative proteomic approaches to correlate variations in the relative abundances and modification states of different r-protein paralogs across tissues, cell types, subcellular-fractions, developmental stages, and environmental and genetic perturbations with ribosome properties and processes upstream and downstream of ribosomes. With this goal in mind, Hummel et al. (2012) and colleagues recently demonstrated, through a highly impressive large-scale label-free MS^E^ quantitative proteomic approach, that the paralog composition (particularly in RPS3aA, RPS5A, RPL8B, and RACK1) of *A. thaliana* leaf cytosolic ribosomes responded significantly to sucrose feeding – a treatment that elicits dramatic changes in gene expression. Similar experiments involving different treatments seem likely to reveal even broader ribosome dynamics involving a wider range of r-proteins.

## From Form to Function: Genetic Studies of r-Protein Function in *Arabidopsis*

While the large-scale use of qualitative and quantitative proteomics approaches to study the composition and dynamics of ribosomes will be essential for elucidating their role in plant physiology, unraveling the precise functions of specific r-proteins will also be greatly assisted by functional genetic studies. A considerable number of genetic studies involving the characterization of *A. thaliana* r-protein mutants have already emerged (see Table 3 for a list of studies, mutants, and phenotypes). Together, these studies highlight, perhaps unsurprisingly, the important role of ribosomes and translation in many aspects of plant development (Byrne, 2009). While the leaf abaxialisation phenotypes of the various r-protein/ASYMMETRIC LEAVES double mutants appear to be somewhat qualitatively independent of which particular r-protein gene is disrupted, the relative severity of different phenotypic sub-elements does seem to depend to some degree on the identity of the disrupted r-protein. These observations support the suggestion that different r-proteins do contribute differently to leaf development (Horiguchi et al., 2011).

**Table 3:** **Published *A. thaliana* r-protein mutants and their phenotypes**.

r-Protein (AGI)	Type of mutant	Mutant name	Phenotype	Reference
L5A(At3G25520)	*rpl5A*/*as2* double EMS mutant	*ae6-1 as2-101*	Abaxialised leaves. Increased number of lotus- and needle-like leaves	Yao et al. (2008)
L5A(At3G25520)	*rpl5A*/*as1* double EMS mutant	*ae6-1 as1-101*	Abaxialised leaves. Increased number of lotus- and needle-like leaves	Yao et al. (2008)
L5A(At3G25520)	*rpl5A* single EMS mutant	*ae6-1*	Normal wildtype phenotype	Yao et al. (2008)
L5A(At3g25520)	*rpl5A*/*as1* double EMS mutant	*pgy3*	Dramatic ectopic lamina outgrowths on the adaxial side of the leaf	Pinon et al. (2008)
L5B(At5G39740)	*rpl5B* single EMS mutant	*rpl5b*	Pale green leaves	Yao et al. (2008)
L5B(At5G39740)	*rpl5Bas2-101* double EMS mutant	*rpl5b as2-101*	Abaxialised leaves. Almost all leaves needle-like	Yao et al. (2008)
L9C(At1g33140)	*rpl9C*/*as1* double EMS mutant	*pgy2*	Dramatic ectopic lamina outgrowths on the adaxial side of the leaf	Pinon et al. (2008)
L10A(At1G14320)	*rpl10A* homozygous knockout		Lethal	Ferreyra et al. (2010b)
L10A(At1G14320)	heterozygous T-DNA insertion causing reduced mRNA levels		Conditional translational deficiency under UV-B stress	Ferreyra et al. (2010b)
L10A(At1g14320)	*rpl10a*/*acl5* double EMS mutant	*acl5-1*	Semi-dominant mutation in *L10A* rescues the severe dwarf phenotype resulting from mutation of acl5 which encodes thermospermine synthase	Imai et al. (2008)
L10B(At1G26910)	heterozygous T-DNA insertion causing reduced mRNA levels		Abnormal growth including reduced size, narrow, and pointed first leaves, 77% reduction in seedling leaf size, smaller but similar numbers of leaves until flowering time at which point the mutant continued producing leaves and started showing increased rosette branching. Shorter primary roots and reduced silique length were also observed	Ferreyra et al. (2010b)
L10aB(At2g27530)	*rpl10aB*/*as1* double EMS mutant	*piggyback1 (pgy1)*	Dramatic ectopic lamina outgrowths on the adaxial side of the leaf	Pinon et al. (2008)
L23aA(At2G39460)	RNAi knockdown		Growth retardation, irregular root and leaf morphology, abnormal phyllotaxy, and vasculature and loss of apical dominance	Degenhardt and Bonham-Smith (2008)
L23aB(At3G55280)	RNAi knockdown		No visible phenotype	Degenhardt and Bonham-Smith (2008)
L24B(At3G53020)	*rpl24B*/*as2-101* double EMS mutant	*stv1 as2-101*	Abaxialised leaves. Almost all leaves needle-like	Yao et al. (2008)
L24B(At3G53020)	EMS mutant	*short valve (stv1)*	Pale green leaves	Yao et al. (2008)
L24B(At3g53020)	EMS mutant	*short valve (stv1)*	Basal region of ovary shortened. Gynophore elongated	Nishimura et al. (2004, 2005)
L27aC(At1g70600)	*rpl27aC*/*as1* double EMS mutant	*pgy6/rpl27ac-1d*	Altered shoot development, including leaf patterning, inflorescence and floral meristem function, and seed set. A temporal delay in initiation and outgrowth of cotyledon primordia leads to development of an enlarged globular embryo prior to apical domain patterning	Szakonyi and Byrne (2011)
L27aC(At1g70600)	*rpl27aC* knockdown mutant (T-DNA insertion in promoter) – homozygous	*rpl27ac-2*	Pointed and serrated leaves	Szakonyi and Byrne (2011)
L27aC(At1g70600)	*rpl27aC* knockdown mutant (T-DNA insertion in promoter) – heterozygous	*rpl27ac-2/*+	No visible shoot phenotype	Szakonyi and Byrne (2011)
L27aC(At1g70600)	*rpl27aC* knockout mutant (T-DNA insertion in 5′-UTR) – homozygous	*rpl27ac-3*	Pointed and serrated leaves (less so than in *rpl27ac-2*)	Szakonyi and Byrne (2011)
L27aC(At1g70600)	*rpl27aC* knockout mutant (T-DNA insertion in 5′-UTR) – heterozygous	*rpl27ac-3/*+	No visible shoot phenotype	Szakonyi and Byrne (2011)
L28A(At2G19730)	*rpl28A*/*as2* double EMS mutant	*ae5-1 as2-101*	Abaxialised leaves. Increased number of lotus- and needle-like leaves	Yao et al. (2008)
L28A(At2G19730)	*rpl28A*/*as1* double EMS mutant	*ae5-1 as1-101*	Abaxialised leaves. Increased number of lotus- and needle-like leaves	Yao et al. (2008)
L28A(At2G19730)	*rpl28A* single EMS mutant	*ae5-1*	Pale green leaves. First few leaves slightly longer than wildtype	Yao et al. (2008)
S5A(At2g37270)	T-DNA insertion knockout	*Arabidopsis Minute-like 1 (aml1)*	Most cell-division processes delayed or disturbed in the heterozygous mutant. Development is completely arrested at an early embryonic stage in the homozygous mutant	Weijers et al. (2001)
S6B(At5g10360)	Antisense knockdown		Reduced apical dominance and irregular positioning of leaves and flowers	Morimoto et al. (2002)
S10B(At5g41520)	*rps10B-1*/*max2-1* double EMS mutant		Recessive mutation in *S10B* suppresses the excessive branching phenotype of *max2-1*	Stirnberg et al. (2012)
S13A(At3g60770)	Transposon-mediated knockout	*Pointed First Leaf 2 (pfl2)*	Aberrant leaf and trichome morphology, retarded root growth, and late flowering. Reproductive growth otherwise not altered. Reduced number of palisade cells. No phenotypic changes observed when crossed with a S18 mutant, *pfl1*, having a similar phenotype	Ito et al. (2000)
S15aE(At4g29430)	T-DNA insertion knockdown	*rps15aE-mut1*	Greater mean rosette radii and leaf areas and longer roots	Szick-Miranda et al. (2010)
S18A(At1g22780)	T-DNA insertion knockout	*Pointed First Leaf 1 (pfl1)*	Pointed first leaves, reduced fresh weight, and growth retardation	Van Lijsebettens et al. (1994)
S27A(At2g45710)	T-DNA insertion knockout	*rps27A*	Conditional growth inhibition under genotoxic stress (growth on methylmethanesulfonate-containing medium). Impairment in mRNA degradation after UV irradiation	Revenkova et al. (1999)

*EMS, ethylmethanesulfonate; UV, Ultra-violet*.

In addition to the plethora of developmental defects observed in many r-protein mutants, other interesting phenotypes associated with r-protein mutants include the conditional translational deficiency phenotype of L10A mutants exposed the UV-B stress (Ferreyra et al., 2010a,b) and the conditional growth inhibition of S27A mutants grown on genotoxic methyl methane sulfonate-containing medium (Revenkova et al., 1999). Also interesting is a defect in mRNA degradation seen in S27A mutants exposed to UV light (Revenkova et al., 1999).

## Future Strategies to Reverse-Engineer the Physiological Role of Ribosome Heterogeneity

One of the fundamental goals of ribosome research is to understand the role of ribosome heterogeneity in translational specialization and control. Indeed, the fact that different mRNA profiles are associated with polysomes isolated from different cell types (Mustroph et al., 2009) or under different environmental conditions (Branco-Price et al., 2005, 2008; Piques et al., 2009; Liu et al., 2012), combined with the fact that ribosome heterogeneity is also under environmental and developmental control (Szick-Miranda and Bailey-Serres, 2001; Branco-Price et al., 2005, 2008; Turkina et al., 2011; Hummel et al., 2012) suggests that there may well be a link. The existence of so many paralogs of each r-protein family in the genome of *Arabidopsis* means that more than 10^34^ theoretical r-protein combinations could potentially be formed *in-vivo* (Hummel et al., 2012). Such incredible capacity for ribosome heterogeneity makes the notion of a ribosome “code” – whereby different ribosomes are optimized for or dedicated to the translation of specific mRNAs (Komili et al., 2007) – particularly alluring. However, proving the existence or otherwise of a ribosome code will be extremely challenging.

It will probably never be possible to resolve and characterize every single one of the 10^34^ potential ribosomes. However, we may be able to significantly deepen our understanding of the role of changes or differences in ribosome composition in translational specificity by fractionating ribosome and polysome populations, analyzing the fractions by translatomic (Mustroph et al., 2009) and quantitative proteomic (Hummel et al., 2012) approaches and then mining the resulting data for correlations between ribosome composition and translational behavior. The phosphorylation of S6 has already been correlated with differential mRNA recruitment to ribosomes (Scharf and Nover, 1982; Turck et al., 2004). However, global integrated proteomic and translatomic analyses across a much wider range of ribosome types will be essential if we hope to properly decipher the ribosome code and resolve causations from correlations with a high degree of confidence.

The correlative approach described above depends on building up sufficient covariance between ribosome composition and translatome profiles. This variation could be obtained in a wide variety of ways. The quantitative proteomic analysis of polysomes from different cell types for which translatome data are already available (Mustroph et al., 2009) may be a fruitful place to start. However, other possibilities might include the separation of free cytoplasmic polysomes and polysomes bound to various subcellular membrane structures such as the endoplasmic reticulum, mitochondria and chloroplast surfaces (Suissa and Schatz, 1982; Kaltimbacher et al., 2006; Fu et al., 2012). Alternatively, complex polysome populations might be fractionated directly by non-denaturing preparative separation techniques such as free-flow electrophoresis which separates protein complexes and even organelles on the basis of surface charge (Wagner, 1989).

More targeted approaches might include the affinity purification of ribosomes translating “bait” mRNAs containing aptamers enabling their selective immunopurification (along with the ribosomes translating them). Quantitative proteomic comparisons of these ribosomes with those pulled down using control mRNAs might help reveal regulatory elements within test mRNAs that promote their recruitment by polysomes while also revealing the types of ribosomes they attract.

Another targeted approach might be to genetically perturb the expression of particular r-proteins and then monitor and cross-correlate changes in ribosome composition with changes in the translatome. However, given the high potential for pleiotropic effects when disrupting translation machinery, perhaps an appropriate approach would be to employ inducible, possibly cell type-specific, silencing, or over expression of r-proteins so that time-course profiling of the ribosomal proteome and translatome can be used to distinguish primary (early) and secondary (later) effects of specific r-protein perturbations.

Another potentially powerful approach to understand how changes in ribosome composition are related to changes in translation may be to apply next-generation ribosome footprinting whereby the exact locations of ribosomes on transcripts is determined by deep sequencing the regions of transcripts that are protected by ribosomes (Ingolia et al., 2009; Lee et al., 2012). Combining this technique with polysome fractionation, genetic-, and environmental-perturbation and quantitative proteomics may reveal, on a genome-wide scale and with single-base-pair resolution, how ribosome composition is related to mRNA occupancy and, through motif analysis, the affinity of ribosomes for particular mRNA sequence elements.

Clearly, as far as the structures and functions of ribosomes are concerned, there are still many more questions than answers. Despite being discovered so long ago, ribosomes remain one of the most interesting and crucially important targets for basic and applied biological research. However, being the complex natural nano-machines that they are, they do not give up their secrets easily and will no doubt remain the focus of many research careers well into the foreseeable future.

## Conflict of Interest Statement

The authors declare that the research was conducted in the absence of any commercial or financial relationships that could be construed as a potential conflict of interest.

## Supplementary Material

The Supplementary Material for this article can be found online at http://www.frontiersin.org/Plant_Proteomics/10.3389/fpls.2013.00032/abstract
